# Ocular vestibular-evoked myogenic potentials in unilateral Ménière’s disease: a narrative literature review

**DOI:** 10.3389/fneur.2026.1937524

**Published:** 2026-09-30

**Authors:** Mitesh Patel, Carl Walter, Per-Anders Fransson

**Affiliations:** 1 North Wales Medical School, Bangor University, Bangor, United Kingdom; 2 Department of Otorhinolaryngology Head and Neck Surgery, Skåne University Hospital, Lund, Sweden; 3 Department of Clinical Sciences Lund, Lund University, Lund, Sweden

**Keywords:** clinical utility, diagnosis, Ménière’s disease, oVEMP, utricle

## Abstract

The ocular vestibular-evoked myogenic potential (oVEMP) is a promising non-invasive tool for assessing utricular function and superior vestibular nerve integrity. In the context of definite unilateral Ménière’s disease, studies have consistently reported altered oVEMP responses in affected ears, including altered amplitudes, higher stimuli needed before receiving a verifiable response or prolonged latencies. These findings are thought to reflect the presence and accumulation of endolymphatic hydrops. However, variability across studies is substantial. This inconsistency may reflect fluctuating changes in endolymphatic pressure or volume and consequent variability in otolith function that characterise Ménière’s disease symptoms, as well as differences in disease stage and progression, stimulus parameters and recording methodologies. The specificity of oVEMP findings for Ménière’s disease remains limited by variation in stimulus modality (air- versus bone-conducted), stimulus intensity, stimulus settings (e.g., rise and fall time, and duration) and recording protocol. Because oVEMP primarily assesses utricular function, it provides information that complements conventional vestibular tests of other vestibular end organs or neural pathways such as caloric testing, video head impulse testing and cervical VEMP. While oVEMP may contribute to a multimodal assessment of Ménière’s disease, further standardisation is required before its diagnostic value can be reliably compared across clinical and experimental studies. Longitudinal studies are also needed to establish how oVEMP responses change with disease stage, disease progression, and following treatment.

## Introduction

Ménière’s disease is characterised by recurrent episodes of spontaneous vertigo, fluctuating sensorineural hearing loss, tinnitus and aural fullness. The diagnosis of definite Ménière’s disease requires two or more episodic attacks of vertigo associated with low- to medium-frequency sensorineural hearing loss and fluctuating aural symptoms (hearing, tinnitus and/or fullness) in the affected ear, supported by the results of clinical testing and examination. The duration of vertigo episodes in typical cases lasts between 20 min and 12 h (1, 2). The disease is not a single condition and is associated with rare genetic variants in both familial and sporadic cases, with changes in the innate immune response leading to the pathological hallmark of endolymphatic hydrops. Several of the ~20 abnormal genes found encode for structural stereocilia or tectorial membrane proteins, that when impaired could lead to the accumulation of endolymph in the cochlear duct. Hence, molecular changes precede the changes in the regulation of endolymph and perilymph (3, 4). Ménière’s disease also appears to be associated with changes in afferent and efferent nerve signalling, intercellular signalling, metabolism and blood flow (5). Specifically concerning nerve signalling, modulation of vestibular afferent and efferent activity has been proposed as a potential contributor to abnormal vestibular responsiveness, while dysfunction of the olivocochlear efferent system may be relevant primarily to cochlear manifestations (6). Research has shown that vestibular hair cells receive cholinergic efferent nerve innervation, which can modulate hair-cell and primary afferent activity, thereby influencing the gain and dynamic range of vestibular signalling. Alterations in this modulatory system have therefore been proposed as a potential contributor to abnormal vestibular responsiveness (7).

The pathological hallmark of Ménière’s disease is the development of endolymphatic hydrops and expansion of the endolymphatic space, thought to arise from distention of Reissner’s membrane (8). Expansion of the endolymphatic space may be caused by hydrodynamic distension, and possibly by hyperplastic growth, as a recent histological study showed a 4- to 7-fold increase in epithelial cell numbers in Reissner’s and saccular membranes, acting as a compensatory organ-wide response to cell loss in the endolymphatic sac (9). Furthermore, the pars superior and pars inferior anatomical organisation of the labyrinth may help to explain the fluctuating vestibular pathology of Ménière’s disease. Because the pars inferior contains the cochlea and saccule, whereas the pars superior contains the utricle and semicircular canals, changes in endolymphatic pressure or volume in one compartment may be transmitted through the connecting ducts and alter the mechanical operating conditions of the other. Experimental endolymph loading in the Guinea pig labyrinth has demonstrated sudden, reversible changes in utricular sensitivity, which Brown et al. (10) proposed could result from pressure-driven opening of the utriculo-saccular duct and resulting alteration of utricular structure and functional dynamics. Such hydrodynamic changes provide a plausible mechanism for transient alterations in otolith function and, potentially, the enhanced or fluctuating responses observed during earlier or active stages of Ménière’s disease (11). Despite significant advances in vestibular diagnostics, the clinical evaluation of vestibular function in patients with unilateral Ménière’s disease remains challenging, particularly in identifying the degree of otolith organ involvement.

Vestibular evoked myogenic potentials (VEMPs) have emerged as important objective tests for assessing otolith function. Among these, ocular Vestibular Evoked Myogenic Potential (oVEMP) provides a non-invasive electrophysiological measure primarily reflecting utricular function (12) and the integrity of the superior vestibular nerve pathway (12, 13). It should however be pointed out that the superior vestibular nerve is not exclusively utricular. The superior division of the vestibular nerve also carries afferents from the anterosuperior (“hook”) portion of the saccular macula, although the utricular macula is the predominant otolith end-organ and considered the major contributor to the oVEMP response (14). oVEMPs are recorded from the extraocular muscles in response to air-conducted sound (ACS), bone-conducted vibration (BCV) or skull taps stimuli, that assess vestibulo-ocular reflex pathways originating from the utricle. This contrasts with cervical Vestibular Evoked Myogenic Potential (cVEMP), which primarily evaluates saccular function and the inferior vestibular nerve via responses recorded from the sternocleidomastoid muscle (13, 15). Although there is significant evidence of utricular deficiency or augmentation of utricular responses in unilateral Ménière’s disease, the clinical application and diagnostic value of oVEMP remain less extensively studied compared to cVEMP or other modes of vestibular testing such as caloric testing. Structural and functional alterations within the utricle could lead to abnormal oVEMP responses, including reduced or augmented amplitudes, prolonged latencies or absent responses, or where a higher stimulus is needed to produce a valid response. The oVEMP may not only support diagnosis, but it might characterise disease severity and potentially monitor disease progression (16).

By assessing asymmetries in response amplitude between ears, oVEMP may help identify side-specific vestibular dysfunction corresponding to the affected labyrinth (16, 17). Furthermore, correlations between oVEMP abnormalities and disease stage (18), duration or audiovestibular findings may enhance understanding of the pathophysiological mechanisms underlying Ménière’s disease. Given the potential of oVEMP to provide objective data on utricular dysfunction, a literature review focusing on its clinical application in unilateral Meniere’s disease is warranted. This review aims to critically summarise current evidence, identify patterns and limitations, and highlight the potential diagnostic and prognostic value of oVEMP in this patient population.

The review will provide an overview of utricular involvement in unilateral Ménière’s disease; discuss oVEMP methodology including technical considerations and normative data; describe the advantages and limitations of oVEMP to other vestibular assessments and finally suggest future directions for research and clinical application.

### Structural and functional changes in the utricle and saccule from unilateral Meniere’s disease

Unilateral Ménière’s disease is believed to cause structural alterations of the utricle and saccule because of sustained hydrops. Histopathology shows that the saccular membrane can become markedly distended due to endolymphatic pressure (19). In advanced cases, the saccule may expand to such a degree that it comes into physical contact with adjacent structures such as the stapes footplate or the utricle (20). This abnormal expansion can compress the utricle and alter the orientation of the otolithic membrane. Hydrop-related changes can also alter the biomechanical properties of the otolithic membrane by increasing endolymphatic pressure. An increased endolymphatic pressure may alter the sensitivity of hair cells to linear acceleration, while distortion of the macula may cause inappropriate stimulation during normal head movements. These abnormalities presumably contribute to the cardinal symptoms of the disease such as imbalance, motion intolerance and episodic vertigo.

Furthermore, studies using three-dimensional MRI reconstructions and histopathological analysis have shown that vestibular hydrops are frequently found in the saccule earlier and more severely than other vestibular compartments including the utricle, which itself may only have hydrops in around 50% or fewer of affected ears (21–23). Researchers have proposed that the relative vulnerability of the saccule may explain why vestibular symptoms in Ménière’s disease often occur alongside early auditory disturbances, given the close anatomical and developmental relationship between the saccule and cochlea (24, 25). Over time, repeated episodes may lead to progressive vestibular degeneration extending to the utricle (26). Central vestibular compensation may partly reduce the functional symptoms of peripheral dysfunction through neuroplastic changes within the vestibular cortex and bilateral vestibular nuclei and their commissural and cerebellar connections, together with adaptation of vestibulo-ocular, and vestibulospinal reflexes (27, 28). Central compensation involves changes to GABAergic and glycinergic inhibition, intrinsic neuronal excitability and cerebellar fibre modulation, which helps to regulate the vestibular tone imbalance (29). Increased reliance on visual and proprioceptive inputs can further support longer-term recovery of postural control through sensory substitution and re-weighting. However, compensation does not restore peripheral vestibular receptor function which may be repeatedly disturbed in Ménière’s disease by the fluctuating nature of the disease activity.

Moreover, persistent structural damage within the utricle and saccule may contribute to chronic disequilibrium in later stages of the disease, reflecting peripheral vestibular and cortical changes. Degeneration of hair cells within the maculae of the utricle and saccule have been observed in post-mortem histological studies of the temporal bone in patients affected by Ménière’s disease (30). Tsuji et al. (30) found that type II hair cells across the cristae and maculae were significantly reduced, and there was a loss of Scarpa’s ganglion cells, whereas type I hair-cell densities remained in the normal range. However, it should be noted that histological preparation may introduce cellular and ciliary artefacts during the processes of fixation, tissue processing or sectioning. Consequently, these observations of histological changes are best regarded as evidence of an association between Ménière’s disease and vestibular hair-cell pathology rather than definitive evidence of progressive in-vivo hair-cell degeneration. Furthermore, chronic mechanical stress and disrupted ionic homeostasis may contribute to progressive loss of both type I and type II vestibular hair cells (31), neural support cell changes, epithelial thinning and collapse of sensory structures (32).

## Review of methods

### Principles of oVEMP testing

Ocular VEMPs are short-latency positive electromyographic responses recorded from extraocular muscles following vestibular stimulation. Since its introduction in the early 2000s, oVEMPs have become an important non-invasive method for assessing utricular function. The oVEMP response reflects activation of the vestibulo-ocular reflex (VOR) pathway. When a vestibular stimulus, typically a high-intensity acoustic stimulus or bone-conducted vibration is delivered to the skull, it activates otolith afferents in the utricle. Activation signals from the utricle travel through the superior vestibular nerve to the vestibular nuclei in the brainstem and then via crossed projections to the contralateral ocular motor nuclei (13). The resulting reflex contraction occurs mainly in the inferior oblique muscles, producing a measurable myogenic potential beneath the eye contralateral to the stimulated ear (33). During oVEMP recordings, participants are instructed to maintain an upward gaze. Upward gaze produces sustained contraction of the inferior oblique muscles, which increases baseline EMG activity and thereby improve the signal-to-noise ratio and detectability of vestibular evoked responses (34).

The typical oVEMP waveform consists of an initial negative component at approximately 10 ms (denoted n10), followed by a positive component at approximately 15 ms (denoted p15), measured from stimulus onset (35). These peaks are measured relative to the levels at stimulus onset. The terms n10 and p15 describe the conventional physiological polarity nomenclature of the response. However, some electrode montages and amplifier polarity conventions will display the n10 as an upward deflection in the recorded trace despite being designated “negative.” Thus, the n10 and p15 should be interpreted according to the established oVEMP nomenclature and the recording montage, rather than solely from the direction of the displayed waveform. The amplitude of the n10–p15 complex and its latency provide information about vestibular pathway function. A reduced or augmented amplitude, prolonged latency or absence of the response may indicate dysfunction of the utricle, superior vestibular nerve or the associated central pathways (12). Clinically, oVEMP testing is particularly useful for assessing disorders affecting the otolith organs, including Ménière’s disease, vestibular neuritis and superior semicircular canal dehiscence (13, 36).

### Methodological considerations in oVEMP testing

Despite its widespread clinical use, oVEMP testing protocols are not fully standardised and methodological differences exist between research groups and clinical laboratories. These variations involve stimulus type, stimulus parameters, electrode placement, gaze angle and normative data. Such differences can significantly influence response amplitudes and detection rates (12, 13).

#### Stimulus type

The most commonly used stimuli in oVEMP testing are ACS, BCV and skull taps, see Figure 1.

**Figure 1:**
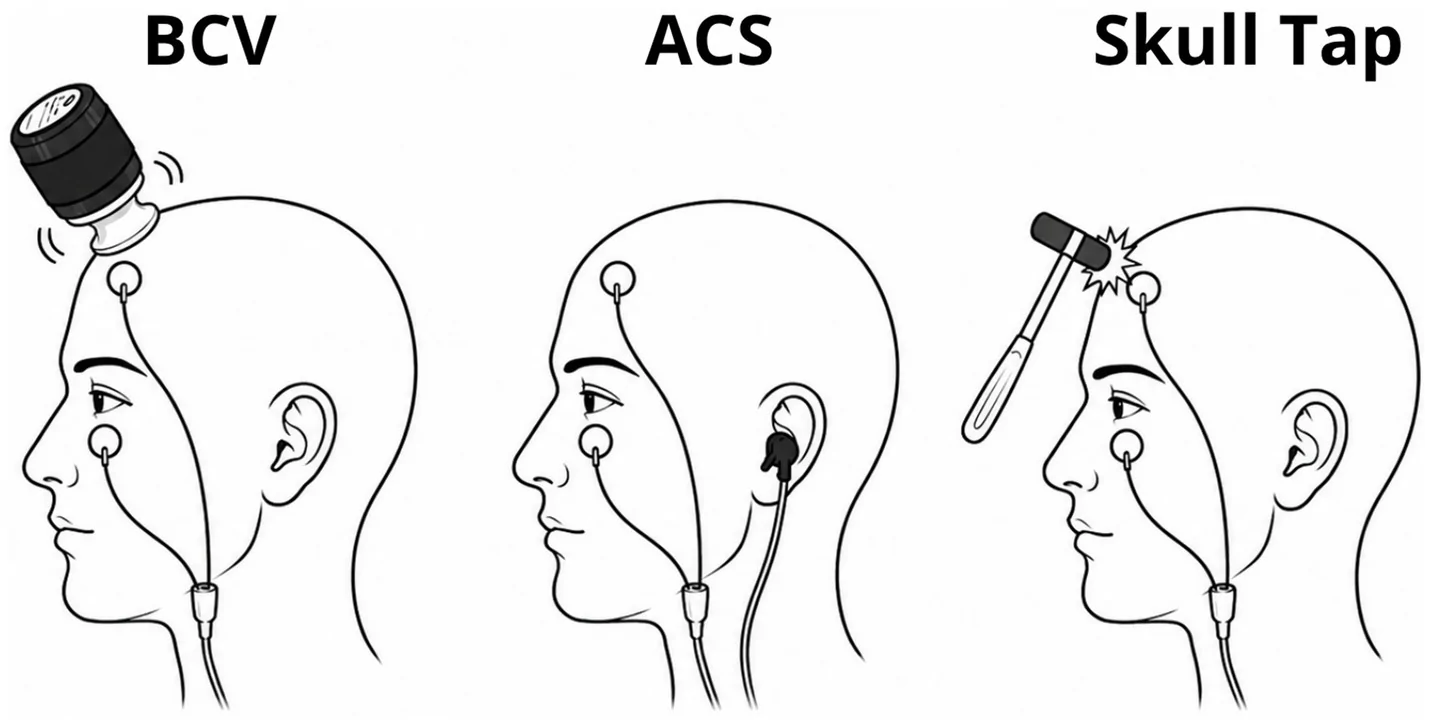
Schematic representation of stimulation modalities used to elicit oVEMPs. Three commonly used stimulation techniques are illustrated. The bone-conducted vibration (BCV) method produces utricular stimulation using vibration transmitted through bone-conduction. In this schematic, the vibrator device (e.g., a B&K 4810, RadioEar B71, RadioEar B81 or B250 transducer) is placed in contact with the forehead while the device elicits burst of vibrations. The air-conducted sound (ACS) method delivers utriculus stimulation through sound elicited by earphones or headphones. Finally, the skull tap method produces utriculus stimulation by performing controlled mechanical taps to the midline forehead, e.g., by using a reflex hammer. In each configuration, the EMG electrodes are positioned over the periorbital region to record the evoked response from the contralateral inferior oblique muscles.

##### Air-conducted sound

ACS stimuli typically consist of tone bursts delivered through insert earphones or headphones. These tone bursts are typically delivered at 500 Hz but 1,000 Hz has also been suggested (37, 38). The duration of the tone bursts used often varies between 2 and 8 ms, but some studies recommend using short tone burst durations (39). ACS stimulation is advantageous because it can be easily implemented using standard auditory evoked potential equipment. However, studies have reported that ACS stimulation produces smaller amplitudes and lower response rates compared with BCV (12). Additionally, ACS responses may be reduced or absent in patients with conductive hearing loss because the acoustic stimulus does not effectively reach the vestibular end organs (13).

##### Bone-conducted vibration

BCV is often considered a more direct and efficient method for activating otolith afferents than ACS. BCV stimuli are typically delivered using a mini-shaker or bone vibrator (e.g., B&K 4810, RadioEar B71, RadioEar B81, B250 transducer) placed on the forehead (Fz position) or mastoid process (12). Rosengren et al. (35) were the first to demonstrate that 8 ms duration bursts of 500 Hz BCV delivered using a RadioEar B71 device reliably elicited oVEMP responses and the evoked responses were altered by pathological conditions including Miller-Fisher syndrome and superior canal dehiscence. This vibration frequency has since then become common in research and clinical BCV protocols (40).

Research by Curthoys and colleagues demonstrated that BCV preferentially activates irregular utricular afferents, producing larger and more reliable oVEMP responses than ACS (12). BCV appears to activate the utricle and saccule relatively equally (41) and may be more specific for utricular examinations when delivered to the mastoid (42). The difference between BCV and ACS appears to be quantitative rather than qualitative, reflecting differences in the magnitude and pattern of peripheral utricular activation (43). Experimental studies by Pastras et al. (44) combining utricular vestibular compound action-potential (vCAP) recordings with simultaneous measurements of macular velocity indicate that the larger response to BCV is primarily peripheral in origin, rather than reflecting stronger activation of downstream utriculo-ocular pathways. Indeed, BCV produces larger utricular vCAPs than ACS even when responses are compared at equivalent macular velocities, indicating modality-dependent differences in the effectiveness of peripheral afferent recruitment. BCV has consequently been widely employed in research and clinical settings when maximising oVEMP amplitude is important.

##### Skull taps

Another method of vestibular stimulation involves manual or mechanical skull taps, typically applied to the midline forehead (Fz) using a reflex hammer or mechanical tapping device. Iwasaki et al. demonstrated that forehead taps can reliably evoke oVEMP responses with short latencies (17). Furthermore, MacDougall et al. (45) demonstrated that oVEMPs can be elicited using a tendon hammer coupled to a smartphone or tablet, supporting the potential utility of this as a low-cost approach. While skull taps are relatively simple to administer, stimulus intensity can vary depending on the examiner, potentially reducing reproducibility. For this reason, many laboratories prefer mechanical vibrators.

#### Stimulus settings

Stimulus settings can vary across studies and may influence both response amplitude and latency.

##### Frequency content in stimulus burst and repetition rate of bursts

For ACS, sound frequencies of between 500 and 1,000 Hz can produce large and reproducible oVEMPs (37), with 500 Hz a commonly used frequency in clinical protocols (40). However, responses can also be elicited at lower frequencies, including 125 and 250 Hz. Frequency tuning is also strongly dependent on stimulus modality. For example, Todd et al. (46) reported band-pass tuning for ACS with the largest responses between approximately 400 and 800 Hz, whereas BCV produced low-pass tuning with the largest responses at approximately 100 Hz. This suggests that oVEMP frequency selection should be considered in relation to the stimulus modality. Higher frequencies such as 750 or 1,000 Hz have also been investigated to characterise vestibular frequency tuning and may provide useful diagnostic information (36, 47). Stimulus repetition rates typically range between 3 and 5 Hz, although slower rates may be used to improve recording stability (13).

##### Stimulus intensity

Research has shown that stimulation intensity can affect oVEMP response amplitude, latency, and detection of features (48, 49). Increasing stimulus intensity above the threshold tends to increase both the amplitude and probability of eliciting an oVEMP response. However, as signal strength and response properties are affected by the type of stimulation (50), results obtained using ACS, BCV, and skull taps are not directly comparable and should be interpreted in the context of stimulus intensity [see Figure 2, for graphical illustrations of the role of stimulation intensity we recommend Xu et al. (51) and Holmeslet et al. (52)]. This is important because these stimulation types are expressed with different units and are not equivalent in terms of the actual degree of mechanical stimulus transmitted to the utricle for the same frequency (53). ACS is routinely reported in terms of dB nHL, dB seSPL, or dB peSPL, whereas BCV is generally expressed as peak force level (dB peVFL/dB FL). For ACS, the measured peak-to-peak equivalent sound pressure level (peSPL) is often referenced to 20 μPa of the air-conduction sound level, whereas for BCV, the measured peak-to-peak vibratory force level (peVFL) is often referenced to 1 μN of the vibratory force level (51).

**Figure 2:**
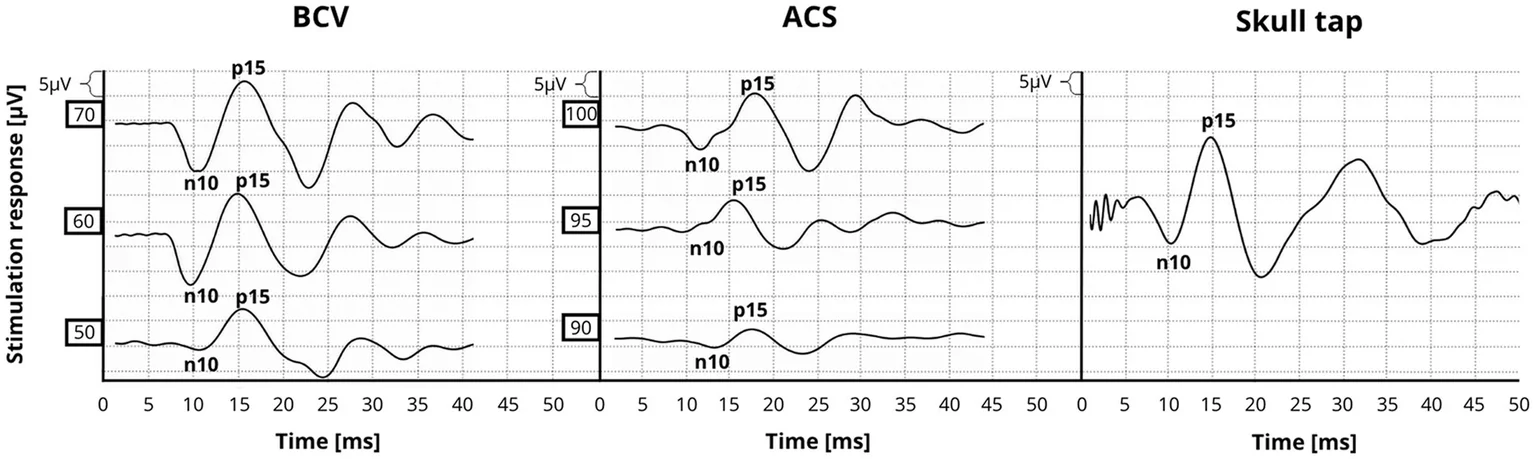
The experimental setup and representative oVEMP waveforms elicited for bone-conducted vibration (BCV); air-conducted sound (ACS), and skull tap. The corresponding oVEMP traces illustrate the characteristic early n10 and p15 components, with stimulus intensity/level indicated alongside the individual traces. For BCV and ACS, recordings obtained at different stimulus levels are shown [BCV: 50, 60, and 70 dB peVFL; ACS: 90, 95, and 100 dB peSPL tone bursts, in line with routine stimulus-level conventions (51)]. Waveforms are displayed over a 0–50 ms recording window, with the amplitude scale indicated in μV. Traces show higher response amplitudes for BCV than for ACS, and higher response amplitudes for higher intensities of stimulation, reflecting typical physiological responses. Skull taps have a vibration component, and the response amplitude is affected by the location of the skull tap (52). The n10 and p15 labels denote the conventional oVEMP response components and should be interpreted according to their electrophysiological polarity rather than solely by the visual direction of waveform deflection. Differences in displayed polarity may arise from the electrode montage and amplifier polarity convention. Consequently, an n10 may appear as an upward rather than downward deflection in a recorded trace.

According to Rosengren et al. (38), the optimum stimulation intensity for ACS is close to the limits for safe sound exposure, with the average threshold for an oVEMP at around 114 dB peSPL (or between 95 and 105 dB nHL). Hence, increasing the intensity of ACS to establish a signal comparable to BCV for equivalent stimulus parameters could lead to patient discomfort and exposure to unsafe levels of sound (54).

For skull taps, the relationship between nominal stimulus level and acceleration is determined by the force transmitted between the skull and a force transducer. Although midline skull taps produce reproducible oVEMPs that are sensitive to unilateral vestibular loss (55), changes to the skull tapping site and direction can alter signal amplitude, latency and polarity (56, 57). For example, occipital skull taps may produce particularly large responses compared to forehead and vertex (56). Furthermore, the degree of direct end-organ utricular stimulation is related to tap strength and the intrinsic properties of the skull (56, 58, 59). This is important because skull taps produce vibration stimulation and a direct mechanical perturbation (60). Consequently, oVEMP responses elicited by skull taps can be highly variable and are not uniformly larger or smaller than those elicited by ACS or BCV tone bursts. Therefore, signal standardisation is required, and skull-tap responses are not directly comparable with those obtained using ACS, or BCV.

##### Stimulus rise/fall times and stimulus duration

From both a clinical and methodological perspective, rise and fall times of the stimulus should be regarded as an important source of heterogeneity in clinical oVEMP testing and in oVEMP experimental studies. Stimulus rise and fall times are a relatively underreported determinant of oVEMP amplitude and latency and should be considered separately from other measures such as stimulus frequency, intensity and duration. Rise time refers to the interval over which the stimulus increases from baseline to its maximum amplitude, while the fall time describes the return to baseline. The rise and fall time determine the abruptness of stimulus onset and cessation and therefore substantially influences the transient mechanical stimulus delivered to the utricle. Rise and fall times of 1–2 ms with plateau durations of 2 ms are commonly used. However, there are variations, as some studies employ 0-2-0 ms tone bursts (i.e., as rise-plateau-fall times) and others use 1-2-1 ms or 2-2-2 ms envelopes, see Figure 3.

**Figure 3:**
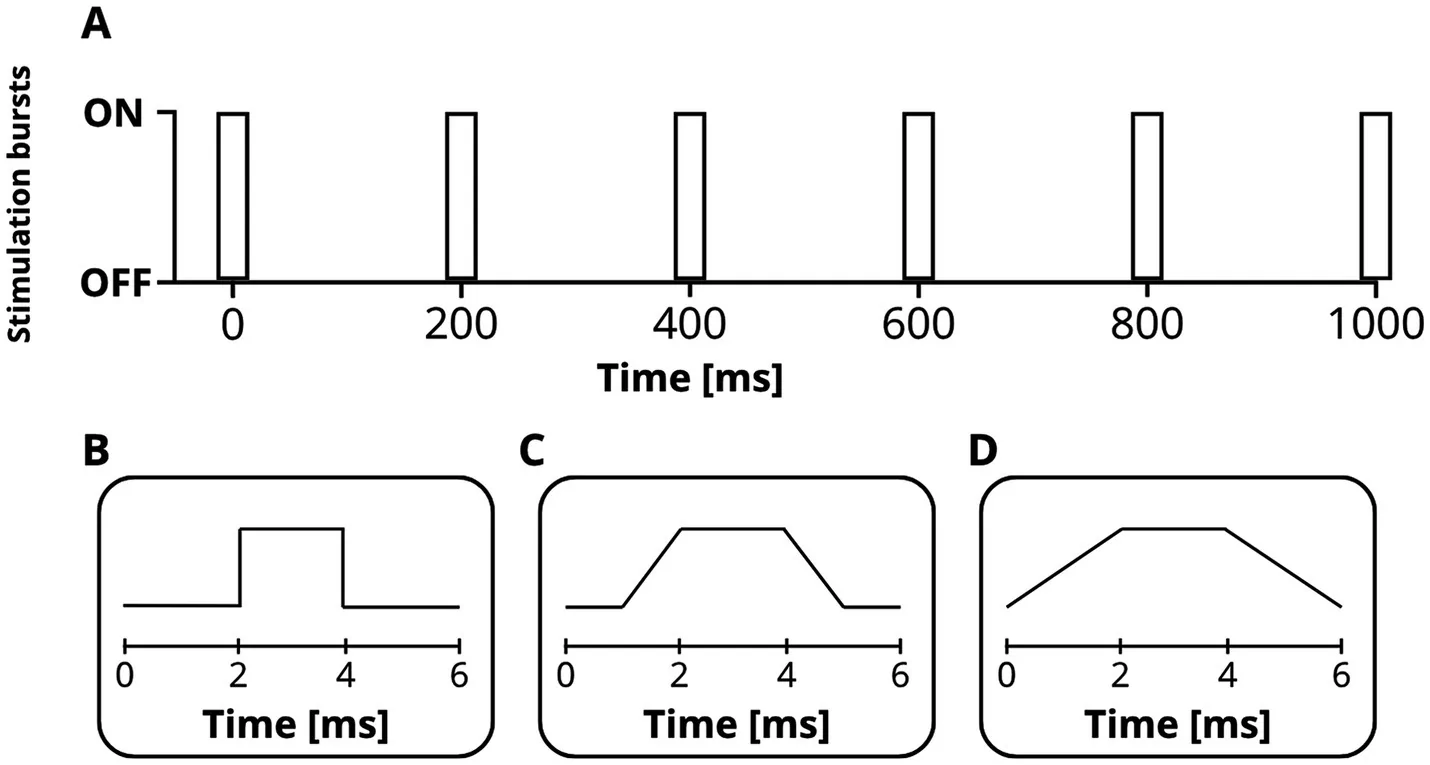
Schematic representation of temporal stimulus repetition and tone burst envelopes typically used in oVEMP recordings. **(A)** Shows a typical-temporal stimulation protocol depicting the repetitive delivery of acoustic or bone-conducted vestibular stimuli over a 1,000 ms window. Stimuli are presented at a standard repetition rate of 5 Hz (equivalent to a 200 ms inter-stimulus interval) to permit adequate myogenic response acquisition **(B)** shows a 0–2–0 ms stimulus envelope (0 ms rise, 2 ms plateau, 0 ms fall). The onset and offset transitions are instantaneous, maximising acoustic energy onset. **(C)** Shows a 1–2–1 ms stimulus envelope. There are a linear 1 ms rise/fall ramp time and a stable 2 ms plateau duration. **(D)** Shows a prolonged 2–2–2 ms gating envelope. The extended tone burst has a 2 ms linear rise/fall ramp time paired with a 2 ms plateau phase. A 0 ms rise time is generally recommended when evoking an oVEMP.

The physiological basis for this effect is becoming more evident. Otolith afferents, particularly the irregular afferents associated with the striola and type I hair cells, are highly responsive to transient stimulation and synchronise their discharge to the onset of sound or vibration (61). As described below, computational and experimental studies have indicated that the mechanical stimulus delivered to the utricular macula highly depends on the temporal characteristics of the stimulus. Pastras et al. (44) developed a mathematical model linking sound- and vibration-induced macular motion to the vCAP generation and demonstrated that rapid changes in the stimulus onset produce the mechanical conditions required for synchronised activation of vestibular afferents. Subsequent experimental measurements in the guinea pig utricle within this same research paper showed that when the input voltage was held constant, increasing BCV rise time from 0 to 2 ms progressively reduced both macular velocity and vCAP amplitude, with vCAP amplitude reducing in proportion to the reduction in macular velocity. These findings provide a physiological explanation for why apparently similar oVEMP stimuli can produce substantially different responses when their temporal envelopes differ.

Importantly, this effect is also evident in human VEMP recordings. Burgess et al. (62) investigated the effect of stimulus rise time on the BCV-evoked oVEMP. Six millisecond tone bursts at a range of frequencies from 50 to 1,200 Hz were delivered with rise times ranging from 0 to 2 ms. Burgess et al. found that at low frequencies (e.g., 100 Hz), shorter stimulus rise-times had a small, modest, effect on the n10, whereas at higher frequencies (e.g., 750 Hz), a shorter rise-time increased the n10 amplitude substantially (62). With a 0 ms rise, the n10 amplitude was significantly larger at a lower frequency, with the largest n10 amplitude being between 250 and 500 Hz. The authors concluded that 0 ms rise time with low frequency stimulation was the most effective stimulus for eliciting oVEMP n10 to BCV.

Studies using ACS further support this interpretation. Kantner et al. (63) compared 500-Hz tone bursts with rise–plateau–fall configurations of 0-4-0, 4-0-4, 2-2-2 and 2-4-2 ms. Although all stimulus configurations produced oVEMPs in healthy participants, the 4-0-4 ms stimulus produced the smallest amplitudes, while the shorter rise time configurations (e.g., 2-2-2 and 0-4-0 ms) produced larger n10 amplitude responses. Latencies were also influenced by the temporal envelope, with the shortest rise time stimulus, 0-4-0, producing earlier responses. Although a short rise time of 0 ms is ideal, Kantner et al., go on to recommend the 2-2-2 ms stimulus for ACS, which generated reproduceable oVEMPs at a safe level of acoustic energy exposure.

These observations have been extended to high-frequency oVEMP testing. Curthoys et al. (64) examined oVEMP n10 responses to 4,000 Hz ACS and BCV stimuli using rise times of 0, 1 and 2 ms in patients with CT-verified superior canal dehiscence. Increasing the rise time reduced the oVEMP n10 amplitude for both stimulus modalities. The authors concluded that short rise times are preferable for clinical VEMP testing (recommending 0 ms), particularly when the objective is to maximise the sensitivity of high-frequency oVEMP responses. Collectively, these findings have an important methodological implication for oVEMP studies and interpreting their results. Two studies may both describe their stimulus as having a “500 Hz tone burst” at the same intensity and duration while nevertheless delivering substantially different effective vestibular stimuli if one uses a 0 ms rise time and the other uses 2 ms or 4 ms ramps. The difference is particularly relevant for short-duration stimuli, where a substantial proportion of the stimulus energy is concentrated at onset and offset. Consequently, stimulus duration alone is insufficient to characterise the temporal properties of an oVEMP stimulus.

This issue is particularly pertinent when oVEMP testing is performed with commercially produced evoked-potential or auditory brainstem response (ABR) systems. Many laboratories use equipment originally configured for ABR recordings, with stimulus templates and hardware designed primarily around conventional auditory evoked-potential paradigms. Depending on the system, the user may have limited control over the rise and fall time of the stimulus or may be restricted to predefined tone-burst envelopes. This creates a potential source of methodological heterogeneity that may not be apparent from the description of the stimulus. A study reporting a “500 Hz tone burst” without specifying its rise and fall time, therefore does not necessarily provide enough information to reproduce the vestibular stimulus accurately. The distinction is particularly important because the temporal envelope can affect other stimulus parameters. For example, Curthoys et al. (64) observed a stronger effect of rise time at higher frequencies (4,000 Hz) for both ACS and BCV. The mechanical coupling between the transducer and skull or ear can also modify the actual acceleration, velocity and spectral content delivered to the otolith organs. The electrical or acoustic stimulus waveform should therefore not automatically be assumed to represent the mechanical stimulus reaching the utricle. This is particularly relevant when comparing studies using different BCV transducers or stimuli, because differences in waveform generation and rise time control may introduce response variability.

On stimulus duration, Rosengren et al., stated that increasing stimulus duration tends to increase oVEMP response amplitude (38). However, others have shown that as stimulus duration increased beyond 2 ms, the amplitude of the response decreases (65).

#### Recording techniques

Electrode placement and recording configuration are also critical for obtaining reliable oVEMP recordings. The most commonly used electrode montage involves the active electrode approximately 1 cm below the lower eyelid, reference electrode positioned slightly inferior to the active electrode or on the cheek and ground electrode placed on the forehead (Fz). This configuration records activity primarily from the inferior oblique muscle, which contributes significantly to the oVEMP response (33).

Participants are typically instructed to maintain upward gaze toward a fixed visual target positioned above eye level. This upward gaze increases tone of the inferior oblique muscles, and thus, increases response amplitude (34). However, the recommended gaze angle varies between studies. Rosengren et al. (38) recommend approximately 30° upward gaze though 20–25° gaze angles can be used. Differences in gaze angle could significantly influence amplitude measurements because oVEMPs are dependent on the level of extraocular muscle contraction (66). Noise in oVEMP recordings can originate from movements of the periocular and facial muscles, which includes the orbicularis oculi and the masseter (38, 67). It is therefore important that patients being assessed relax their face and do not clench their jaw.

Signal averaging is generally performed over 100–200 repetitions, and filter settings typically range from 1 to 1,000 Hz or 10 to 1,000 Hz (13). Because the oVEMP pathway is predominantly crossed, the largest response is usually recorded contralateral to the stimulated ear (13). Some laboratories therefore focus on contralateral recordings, while others record responses bilaterally for comparison.

#### Normative data

Normative values for oVEMP responses vary across studies due to differences in stimulus parameters, equipment and participant/patient characteristics. Typical values reported in healthy adults include an n10 latency of between 9 and 12 ms and p15 latency of between 14 and 17 ms (13). Amplitude values show much greater variability. Factors influencing amplitude include gaze elevation, stimulus intensity, stimulus rise/fall times, electrode placement and individual anatomical differences (38, 62). For this reason, many clinicians rely on interaural amplitude asymmetry ratios rather than absolute amplitude values. Asymmetry ratios exceeding 30–40% are commonly considered abnormal in established unilateral Ménière’s disease, although equipment and stimulation-specific thresholds are recommended (36).

Age-related changes are also well documented. Studies have reported reduced oVEMP amplitudes and lower response prevalence in older adults, particularly in individuals over 60 years of age (50, 68). Consequently, some authors recommend establishing age-specific normative ranges. Gender differences have also been reported, with studies observing slightly larger amplitudes in males, possibly due to anatomical differences in muscle mass (69). However, these characteristics are not consistently reported (70).

## Literature review

### oVEMP findings in unilateral Meniere’s disease

The body of literature investigating oVEMPs in Ménière’s disease consistently demonstrates abnormalities reflecting utricular changes, though the sensitivity and specific patterns vary depending on disease stage and methodology. Broadly, the studies can be grouped into four key domains: (1) amplitude reduction, response absence, or amplitude augmentation (2) frequency tuning abnormalities, (3) disease stage associations, and (4) diagnostic utility and multimodal approaches.

#### Amplitude reduction, absent responses or amplitude augmentation

Early studies (between 2011 and 2014), including Taylor et al. (71), Winters et al. (72), Shin et al. (73), and Walther and Blödow (74), showed that reduced or absent oVEMP responses are common in Ménière’s disease, with abnormality rates ranging from approximately 54 to 73%. Subsequent work continued to support these findings. Kingma & Wit (75) and Sobhy et al. (76) demonstrated reduced amplitudes and increased asymmetry, while more studies such as Kharkheli et al. (18), Maheu et al. (77), and Kim et al. (78) confirmed that an oVEMP amplitude reduction remains a robust and reproducible abnormality. Collectively, these findings suggest that oVEMP amplitude reduction is a core electrophysiological marker of utricular involvement in Ménière’s disease, although it lacks disease specificity.

However, authors including Manzari et al. (11) and Wen et al. (79) have demonstrated that there is a dynamic nature to the VEMP responses in Ménière’s disease. For example, Manzari et al. (11) studied patients with unilateral disease during quiescent periods and acute attacks. During vertigo attacks, BCV-evoked oVEMP amplitudes were augmented, whereas cVEMP amplitudes were reduced, demonstrating a dissociation between utricular and saccular responses associated with the current level of activity of the disease. These findings are consistent with the observation that VEMP amplitudes may be increased or fluctuate in the early or active stages (80), before progressive otolith dysfunction and reduced responses become more prominent with disease duration.

#### Frequency tuning abnormalities

A major development in more recent literature is the identification of frequency tuning shifts, particularly a relative increase in responsiveness at 1000 Hz compared to the typical 500 Hz peak. Several authors such as Salviz et al. (81), Winters et al. (82), and Taylor et al. (83), have demonstrated that Ménière’s disease patients often exhibit a shift toward higher-frequency tuning with 1,000 Hz dominance, which is thought to reflect biomechanical changes from hydrops in the utricle. This frequency shift is a specific oVEMP abnormality in Ménière’s disease, that appears to distinguish it from other vestibular disorders such as benign paroxysmal positional vertigo (84).

#### Association with disease stage and severity

Several studies have demonstrated that oVEMP abnormalities correlate with disease progression and severity, and that these abnormalities are particularly evident in patients who experience Tumarkin’s otolithic crises (80, 85, 86). Others have found no association between the degree of hydrops and oVEMP responses (87). Makhoul et al. (16) reported that abnormal oVEMPs are more frequent in late-stage Ménière’s disease, while Chang et al. (88) and Lucieer et al. (89) showed that abnormalities increase with disease duration and are more pronounced in bilateral disease. Jiang et al. (90) further linked abnormal ACS-generated oVEMP responses with worse hearing thresholds, suggesting an association with overall disease burden. These findings indicate that oVEMP abnormalities may evolve over time, potentially reflecting progressive utricular damage.

#### Diagnostic utility and multimodal approaches

Although oVEMP abnormalities are common in Ménière’s disease, multiple studies note that oVEMP is generally less sensitive than cVEMP (91, 92). However, oVEMP may provide complementary information, particularly on utricular function. More recent studies have focused on improving diagnostic performance through combined approaches. Yang and Young (93) demonstrated improved diagnostic accuracy when oVEMP was used alongside cVEMP, whereas Kahn et al. (94) showed that combining oVEMP findings with MRI-based endolymphatic hydrops imaging enhances diagnostic confidence.

Additionally, more recent studies increasingly support the use of frequency tuning (especially 1,000 Hz dominance) as a diagnostic biomarker, rather than relying solely on amplitude or presence/absence/augmentation of responses. Tang et al. (95) and Singh and Barman (84) suggest that incorporating tuning characteristics can improve differentiation between Ménière’s disease and other vestibular disorders. Furthermore, others have confirmed the diagnostic importance of the general shift in the pattern of VEMP responses to higher frequencies of stimulation in Meniere’s disease [for reviews please see Maheu et al. (77) and Makhoul et al. (16)].

### Comparison with other vestibular assessments

Ocular VEMP has emerged as an important tool in the assessment of vestibular function in unilateral Ménière’s disease, offering insight into utricular integrity and superior vestibular nerve pathways. When interpreted alongside established vestibular assessments such as caloric testing, the vHIT and cVEMP, oVEMP contributes to a multidimensional understanding of vestibular dysfunction. However, one of the most defining characteristics of vestibular assessment is the frequent inconsistency between these tests. While certain patterns of agreement exist, particularly in advanced disease, there is discordance between oVEMP and other measures and this may reflect the complex, heterogeneous and frequency-dependent pathophysiology of the disease (96, 97).

In unilateral Ménière’s disease, the disease pathology does not affect all vestibular end organs uniformly. Instead, the semicircular canals and otolith organs may be affected to varying degrees and at different stages of disease progression (98). This selective involvement is likely to be a key factor explaining the variability observed in oVEMP. However, recent research supports the clinical relevance of oVEMP in Ménière’s disease, with one study reporting that oVEMP demonstrated the highest sensitivity among vestibular tests at approximately 68%, compared to 54% for caloric testing and only 7% for vHIT (99).

Several studies have identified a correlation between oVEMP and caloric testing (100–102), which is concordant with the neuroanatomical relationship between the horizontal semicircular canal and utricle which transmit information to the vestibular nucleus predominantly via the superior vestibular nerve (103). Studies have also demonstrated that abnormalities in these tests do not necessarily correlate, with Ménière’s disease patients sometimes exhibiting abnormal oVEMP responses despite normal caloric findings, or vice versa (104). Hence, cVEMP, oVEMP, and caloric tests may provide different topological information about vestibular impairment (105). The caloric test evaluates vestibular function at extremely low frequencies, whereas oVEMP assesses high-frequency utricular responses, reflecting the fundamentally different physiological mechanisms underlying each test.

### Utility of oVEMP in Ménière’s disease

The studies reviewed generally demonstrate that patients with Ménière’s disease exhibit altered oVEMP responses compared to healthy controls, reflecting utricular involvement in the disease. For example, the early electrophysiological study by Taylor and colleagues reported that approximately 50% of patients with definite unilateral Ménière’s disease demonstrated abnormal oVEMP responses when elicited using ACS stimuli, indicating a high prevalence of utricular dysfunction in this population (71).

One of the key strengths of oVEMP lies in its relatively high consistency of showing altered responses in Ménière’s disease. A meta-analysis evaluating the diagnostic value of VEMP (including both oVEMP and cVEMP) in endolymphatic hydrops reported a pooled specificity of approximately 95%, indicating that abnormal VEMP findings are strongly associated with the presence of disease (79, 106). This high specificity suggests that when oVEMP is abnormal, it provides strong confirmatory evidence of vestibular dysfunction consistent with Ménière’s disease. However, the same meta-analysis reported a pooled sensitivity of only 49%, highlighting a significant limitation where a normal oVEMP result does not exclude the presence of Ménière’s disease.

As described previously, further support for the diagnostic utility of oVEMP comes from studies examining frequency tuning characteristics. In healthy individuals, oVEMP responses are typically maximal at 500 Hz. In contrast, patients with Ménière’s disease often exhibit a shift in frequency tuning toward higher frequencies, such as 1,000 Hz (81–83). Interestingly, a study investigating this phenomenon reported that the presence of a frequency tuning shift could distinguish Ménière’s disease from benign paroxysmal positional vertigo (BPPV) with a sensitivity of 68% and a specificity of 100% (84). Such parameters go beyond simple latency, amplitude or interaural asymmetry ratios.

Despite these promising outcomes, the diagnostic utility of oVEMP remains constrained by several issues. Across studies, sensitivity values for VEMP testing in Ménière’s disease generally range from approximately 37 to 64%, depending on the stimulus parameters and patient population. This variability may be attributed to differences in disease stage, testing protocols and criteria for abnormality. In early-stage Ménière’s disease, for example, oVEMP responses may be augmented rather than reduced, reflecting increased sensitivity of the utricle during acute hydropic episodes (11, 79). Lee et al. (80) found that oVEMP responses were more frequently augmented in early-stage disease or during symptomatic attacks whereas reduced or absent responses were more common in later stages. This dynamic behaviour complicates the interpretation of oVEMP findings and may contribute to inconsistent diagnostic performance.

Moreover, oVEMP may have value in detecting subclinical or contralateral involvement. Research has shown that oVEMP abnormalities can occasionally be observed in the clinically unaffected ear of patients with unilateral Ménière’s disease, albeit to a lesser degree (82, 107). This may indicate progression to bilateral disease. The ability of oVEMP to identify such subclinical changes may have prognostic implications as bilateral involvement is associated with a more severe disease course and greater functional impairment. The use of interaural asymmetry ratios can also improve the detection of unilateral abnormalities by accounting for inter-individual variability.

## Discussion

### Clinical application of oVEMP

While oVEMP offers a targeted assessment of utricular function, its outcome must be interpreted in the context of a broader vestibular test battery. The available literature consistently demonstrates that oVEMP possesses relatively high specificity but modest sensitivity, resulting in a positive indicator of disease when abnormal, but it is less effective at ruling out disease when normal.

A key body of evidence comes from meta-analytic data that reported a pooled sensitivity of approximately 49% and a specificity of 95%, indicating that while VEMP abnormalities are highly suggestive of disease, nearly half of affected individuals may present with normal results (106). The study reported that an abnormal VEMP result substantially increases the probability of Ménière’s disease being present (106). Conversely, the study reported that a normal result only modestly reduces disease probability, reinforcing the limitation of oVEMP as a rule-out test (106).

When focusing specifically on oVEMP parameters, diagnostic accuracy may be improved through more refined metrics such as frequency tuning. In healthy individuals, oVEMP responses are typically maximal at 500 Hz, whereas in Ménière’s disease, a shift toward higher frequencies (e.g., 1,000 Hz) is often observed due to hydropic alterations in inner ear mechanics. The specificity implies that such abnormal tuning is indicative of pathological utricular involvement and therefore may be particularly useful in confirming the affected ear in unilateral cases. However, other authors have argued that there does not appear to be a best frequency as there is significant individual variability [e.g., Sandhu et al. (47) showed different oVEMP responses for sound-conducted responses between 750 and 2000 Hz].

Interaural amplitude asymmetry ratios have been proposed as a means of improving the identification of the affected ear by comparing amplitude differences between sides. Elevated asymmetry values may indicate unilateral dysfunction. However, variability in normative thresholds and inter-individual differences, and augmented responses in the acute phase, or in early stages of the disease, complicates the establishment of universally applicable cut-off values. Furthermore, bilateral or subclinical involvement in Ménière’s disease can reduce asymmetry, thereby diminishing the discriminative power of oVEMP.

Beyond its diagnostic contribution, oVEMP could also have potential in monitoring disease progression and response to therapy. The underlying rationale is that oVEMP reflects functional integrity of the utricle, which may deteriorate over time as endolymphatic hydrops progresses. Several studies have demonstrated that oVEMP abnormalities become more prevalent and more severe with increasing disease duration (18, 108). Specifically, reduced amplitudes and absent responses are more commonly observed in patients with longstanding or advanced Ménière’s disease. This progression is consistent with known pathophysiological mechanisms. Endolymphatic hydrops initially causes mechanical distortion of the membranous labyrinth, potentially leading to altered sensitivity and even augmented responses. However, as the disease advances, structural degeneration of the utricular sensory epithelium and vestibular afferents occurs, resulting in diminished oVEMP responses. Experimental and clinical studies support this model, demonstrating that hydropic changes can lead to mechanical damping and reduced neural activation in the utricle (109). Consequently, periodic oVEMP follow-up testing may provide insight into the trajectory of vestibular dysfunction or success of ablative treatment over time.

### Limitations of current evidence

One of the most prominent limitations is the considerable heterogeneity in study populations. Ménière’s disease itself is a clinically heterogeneous condition, with variability in symptom profile, progression and perhaps the underlying pathology. However, this variability is often compounded by differences in inclusion criteria across studies. Some investigations include only patients with definite unilateral Ménière’s disease, while others incorporate probable, or even suspected cases. Additionally, the presence of bilateral disease or subclinical contralateral involvement is not always consistently accounted for or investigated. This lack of uniformity in patient selection introduces variability that complicates comparisons.

Another major limitation lies in the heterogeneity of oVEMP testing protocols. There is currently no universally standardised method for eliciting or recording oVEMP responses, and studies differ in several key methodological parameters. These include the type of stimulus used (ACS versus BCV), stimulus frequency and intensity, duration and rise/fall time of the stimulus, recording electrode placement, patient positioning and criteria for defining abnormality. For example, some studies rely primarily on amplitude measures, while others emphasise latency, interaural asymmetry ratios or frequency tuning characteristics. oVEMP can also produce false-positives with the unaffected ear, which may not be associated with bilateral involvement, demonstrating reduced responsivity (72). The absence of standardisation means that results from different studies are not always directly comparable, and variations in methodology may significantly influence outcome.

This methodological variability also contributes to inconsistencies in reported sensitivity and specificity. Studies using more sensitive stimuli, such as BCV, may report higher detection rates of oVEMP responses (53), while those using ACS may observe higher rates of absent responses (110). Similarly, differences in threshold criteria for abnormality can lead to variations in clinical effectiveness and reported prevalence. Without agreed normative data and standardised procedures and cut-offs, it remains challenging to determine whether differences between studies reflect true physiological variation or methodological variations.

Conflicting results across the literature further highlight the limitations of current evidence. While many studies have reported reduced or absent oVEMP responses in affected ears, others describe preserved or even enhanced responses, particularly in an active early disease stage (11). These discrepancies may reflect genuine differences in disease stage or pathophysiological mechanisms, but they also raise questions about the reliability and reproducibility of oVEMP findings. In some cases, studies have reported significant abnormalities in contralateral ears, suggesting bilateral involvement, while others have found changes to be predominantly unilateral. Similarly, while altered frequency tuning is often described as a characteristic feature of Ménière’s disease, its prevalence and specificity vary between studies. These inconsistencies make it difficult to establish clear diagnostic criteria or to define the precise role of oVEMP in clinical practise.

A further limitation is the cross-sectional design of most studies. Longitudinal data on oVEMP changes in Ménière’s disease are relatively scarce, limiting understanding of how utricular function evolves with disease progression. Without longitudinal studies, it is difficult to determine whether observed abnormalities represent transient changes with disease activity, progressive deterioration or stable deficits that have little bearing on functional outcomes. This gap in knowledge also restricts the ability to evaluate the prognostic value of oVEMP or its usefulness in monitoring treatment response. While some studies suggest that oVEMP abnormalities increase with disease duration, the lack of consistent longitudinal evidence means that these conclusions remain tentative.

Whilst oVEMP has gained promise as a relatively accessible measure of utricular function, a number of additional techniques, have been used. The subjective visual vertical (SVV) is among the most commonly used clinical tools in vestibular testing and is presumed to examine the utricle’s role in encoding head orientation relative to gravity. Deviations in SVV are well documented in acute unilateral vestibular lesions, typically toward the affected side. However, its diagnostic specificity is limited by central compensation and visual dependence (111), and the SVV is likely to be influenced by cervical proprioception and semicircular canal function (112). Furthermore, the SVV may be positive in acute settings (113, 114), but not in chronic deficiency. More direct but less accessible approaches include unilateral centrifugation, which selectively stimulates each utricle through eccentric rotation and can quantify asymmetries in otolith function, though its clinical utility is constrained by the need for specialised equipment and limited tolerability, but the technique has shown good reliability (115, 116). Psychophysical paradigms such as vestibular perceptual threshold testing have also gained attention for their sensitivity to subtle dysfunction, measuring the minimum detectable linear acceleration presumed to be mediated largely by the utricle, but these methods remain largely confined to research settings due to technical demands and lab-specific normative datasets (117, 118). Finally, experimental paradigms have attempted to isolate utricular contributions by applying controlled linear accelerations while measuring compensatory eye movements, as the translational VOR (tVOR) (119). However, the tVOR is highly dependent on viewing distance, target characteristics and central predictive mechanisms, making it difficult to interpret.

### Future directions

One key area identified is the standardisation of oVEMP testing protocols and parameters. Variability in the techniques employed during the assessment and in the analysis of results has contributed to inconsistent findings across studies. This includes establishing protocols for frequency tuning curves, duration and rise/fall times of the stimulus, stimulation intensity, interaural amplitude asymmetry ratios and threshold testing measures. Establishing universally accepted normative data and thresholds for abnormality would improve interpretation and facilitate comparison between clinical groups.

There is also a requirement to study the role of oVEMP in longitudinal monitoring of Ménière’s disease, with the aim of determining whether these changes correlate with disease progression, symptom fluctuation or treatment outcomes. Such work could help establish oVEMP as a tool for diagnosis and measuring long-term prognosis and treatment outcomes prospectively.

## Conclusion

Ocular VEMP represents a valuable addition to the vestibular test battery in unilateral Ménière’s disease, with particular strengths in detecting utricular dysfunction and contributing to diagnosis. While it demonstrates high specificity, and promising diagnostic features such as frequency tuning shifts, its overall utility is constrained by limited sensitivity and variability across patients. As such, oVEMP should be interpreted within a multimodal framework and in conjunction with clinical findings.
